# Myocardial Expression of Estrogen Receptor-mRNA Is Associated With Lower Markers of Post-operative Organ Damage in Young Patients With Congenital Cardiac Defect

**DOI:** 10.3389/fped.2021.729198

**Published:** 2021-09-22

**Authors:** Hatem Rouatbi, Nesrine Farhat, Ruth Heying, Jaime F. Vazquez-Jimenez, Anne-Simone Parent, Marie-Christine Seghaye

**Affiliations:** ^1^Department of Pediatrics and Pediatric Cardiology, University Hospital Liège, Liège, Belgium; ^2^Department of Pediatric Cardiology, University Hospital Leuven, Leuven, Belgium; ^3^Department of Pediatric and Congenital Cardiac Surgery, University Hospital Aachen, Aachen, Germany; ^4^Department of Pediatric Endocrinology, University Hospital Liège, Liège, Belgium

**Keywords:** ERα, ERβ, cytokines, myocardial expression, myocardial protection

## Abstract

**Background:** Estrogen receptors (ERs) relate to cardio-protection in adults, but their role in younger patients is not known. We aimed to assess the myocardial expression of ERα- and ERβ- mRNA in young patients with congenital cardiac disease and to analyze their putative protective role.

**Patients and Methods:** Twenty children and young adults (seven females and 13 males) with a median age of 13.8 years (interquartile range: 12.3 years) were enrolled in this prospective study. The myocardial expression of ER-mRNA and genes involved in inflammation, growth, and stress response was assessed by real-time PCR and was correlated to post-operative (po) outcome.

**Results:** ER-mRNA was detected in the myocardium of all patients, independently of gender and age. The expression of ER-mRNA correlated with that of mRNA coding for brain natriuretic peptide and for all cytokines tested. A higher ERα-mRNA expression correlated with lower troponin T concentrations at 24 h po (*p* = 0.032), higher PaO_2_/FiO_2_ ratio at 4 h po (*p* = 0.059), lower fluid retention at 4 h po (*p* = 0.048), and lower aspartate aminotransferase (AST) levels at 24 h po (*p* = 0.047). A higher ERβ-mRNA expression was also correlated with lower fluid retention at 24 h po (*p* = 0.048).

Patients in whom the levels of ERα- and ERβ-mRNA were >P50 had lower troponin T (*p* = 0.003, respectively) and lower AST concentrations at 24 h po (*p* = 0.043, respectively) than the others.

**Conclusions:** The expression of ERα- and ERβ-mRNA is present in the myocardium of children and young adults with congenital cardiac defect and is associated with lower markers of po organ damage. This suggests that ERs may provide perioperative organ protection in this population.

## Introduction

Estrogens are pleiotropic steroids with cardio-protective properties (1) that are related to vasodilation, anti-inflammatory, and anti-oxidant effects, inhibition of proliferation, and increased cell survival (2).

The physiological effects of estrogens are mediated by estrogen receptors (ERs) that possess a complex signaling that is not fully understood yet (3). It is admitted that the nuclear receptors ERα and ERβ are responsible for the genomic effects of estrogen and initiate ligand-activated transcription by binding estrogen receptor elements (ERE) to the promoter and regulatory regions of target genes. ERα and ERβ are encoded by two separate genes and have a different distribution within tissues and cells, including circulating cells (4). Both receptors are expressed in cardiomyocytes, smooth muscle cells, and endothelial cells and elicit different actions on the cardiovascular system (5). The ratio of their respective tissue concentrations is thought to play a crucial role in the biological response to estrogen (6). ERα and ERβ are not only located into the nucleus but also in cell membrane caveolae and exert acute effects by activating non-nuclear signaling pathways such as PI3K/Akt kinase and ERK1/2 (7). Besides nuclear receptors, estrogens also bind to a membrane receptor called G-protein coupled ER (GPER) present in cardiomyocytes that initiates rapid non-nuclear signaling (8). Estrogen activity involves a cross-talk and collaboration between nuclear and membrane signaling (9).

It is not known as yet whether the myocardium of children expresses ER, while the brain tissue of pre-pubertal children does. Indeed a role of ERs in the pathophysiology of autism in young children has been suggested (10), implying a ligand-independent activity (11) or the activation of ERs by a variety of exogenous receptor ligands, such as phytoestrogens, metallo-estrogens, and xeno-estrogens (9), that pre-pubertal individuals are naturally exposed to.

In adults, the expression of ERs is increased in pressure-loaded myocardium (12), while in children with congenital cardiac disease, hemodynamic overload induces the expression of genes involved in early stress response, inflammation, apoptosis, and fibrosis (13).

This prompted us to address the question of whether ERs would also be expressed in the myocardium of children and young adults with congenital cardiac defect and would interact with the inflammatory response to hemodynamic overload.

Our study was therefore designed to assess, as primary objective, the expression of mRNA coding for ERα and ERβ in the right atrial myocardium of children and young adults with congenital cardiac defect and to correlate this expression to that of mRNA coding for inflammatory cytokines and markers of myocardial stress involved in the pathophysiology of myocardial remodeling.

The secondary objective was to test the hypothesis that myocardial ER expression would relate to myocardial protection and influence the post-operative outcome.

## Patients and Methods

### Patients

After an approval by the Human Ethical Committee of the Aachen University of Technology and informed consent of the caregivers or the patients, if applicable, 20 consecutive patients (seven females and 13 male) with a median age of 13.8 years (minimum: 3 months, maximum 26.5 years; interquartile range, IQR: 12.3 years) were enrolled in this prospective study. Eight patients were younger than 12 years and pre-pubertal, 12 were older than 12 years and had reached puberty. No patient was on estrogen/progestogen combination. Table 1 summarizes the characteristics of the subjects.

**Table 1:** Patient characteristics.

	**Male ** ***n* = 13**	**Female ** ***n* = 7**
Median age in months [IQR]	144 [98,5]^*^	179 [105,9]^*^
Cardiac defect; *Operative procedure*		
	VSD; *Closure (n* = *4)* AoV stenosis; *Commissurotomy (n* = *4*) MV stenosis; *MV plasty (n* = *1) MV valve replacement (n* = *1)* TAPVR; *Repair (n* = *1)* PV stenosis; *Commissurotomy (n = 1)* Ao root dilation; *Tirone David procedure (n = 1)*	VSD; *Closure (n = 3)*ASD; *Closure (n = 2)*A-P Window; *Closure (n = 1)*PA, VSD; *VSD closure, RVOT reconstruction with homograft implantation (n = 1)*
**Weight (kg)**	51 [30,5]^*^	[57,7]^*^
**Expression of ERα- and ERβ-mRNA**		
**>P50** **<** **P50**	*n =* 8 *n =* 5	*n =* 2 *n =* 5

* *P < 0.05 between both groups. Ao, aortic; A-P, aorto-pulmonary; AoV, aortic valve; ASD, atria septum defect; MV, mitral valve; PV, pulmonary valve; P50, percentile 50; RVOT, right ventricular outflow tract; VSD, ventricular septum defect; TAPVR, total anomalous pulmonary venous return. Results are shown as median value [interquartile range]*.

### Perioperative Monitoring and Therapy

All surgical procedures were performed by the same pediatric cardiac surgeon. In all cases, conventional general anesthesia consisted of isoflurane and sufentanyl. Dexamethasone (1 mg/m^2^ body surface area) was given before the sternotomy. Perioperative antibiotic prophylaxis was carried out with cefuroxime. Before the institution of a cardiopulmonary bypass (CPB), a right atrial biopsy was taken. After the institution of a moderate hypothermic low-flow CPB, the aorta was cross-clamped, and cardiac arrest was instituted by intra-aortal injection of 4 °C cold cardioplegic solution (Bretschneider, 30 ml/kg body weight) that was re-aspirated in the right atrium. After the intra-cardiac repair, the patient was weaned from CPB under progressive re-warming. Epicardiac pacemaker leads and pericardial and mediastinal drains were placed before chest closure.

The arterial blood pressure and central venous pressure were continuously monitored *via* an arterial and a central venous line, respectively.

Inotropic support, which consisted of dobutamine, was given to maintain a normal mean arterial blood pressure for age and volume therapy by injections of crystalloid solutions, if requested. The patient was transported to the intensive care unit where weaning from artificial ventilation was begun as early as possible. The ratio between the arterial partial pressure of oxygen (PaO_2_) and the fraction of inspired oxygen (FiO_2_) was used to assess oxygenation. Diuresis was continuously monitored *via* a bladder catheter, and water balance was calculated hourly.

The routinely performed laboratory investigations included the determination of blood gases, blood concentration of lactate, glycemia, complete blood count, serum creatinine, aspartate aminotransferase (AST), troponin T, and coagulation parameters and were measured at least 4 and 24 h post-operatively.

### Reverse Transcriptase-Polymerase Chain Reaction

Biopsies taken for the detection of messenger ribonucleic acid (mRNA) were immediately snap-frozen in liquid nitrogen and stored at −80 °C until analysis.

Total ribonucleic acid (RNA) was extracted from the atrial myocardium by using the RNeasy kit (QIAGEN Inc., Hilden, Germany). The RNA (100 ng) was reverse-transcribed to complementary deoxyribonucleic acid (DNA) with random hexamers. A 2-μl cDNA sample was incubated with 20 μl of QuantiTect Mix containing fluorescence dye SYBR® Advantage® qPCR premix from Clontech (Takara Bio Inc. Otsu, Shiga, Japan).

The expression of target genes was normalized to the levels of 18S-mRNA and calculated with 2^−ΔCT^. Besides the expression of mRNA coding for ERα and ERβ, the expression of mRNA coding for the pro-inflammatory cytokine tumor necrosis factor-a (TNFα), interleukin (IL)-1β, for the regulator of the acute phase reaction that shares pro- and anti-inflammatory properties, IL-6, for chemokine IL-8, for the anti-inflammatory cytokine IL-10, for the growth factor and major regulator of fibrosis tissue growth factor (TGF)-β, for the main growth factor of cardiomyocytes cardiotrophin (CT)-1, and for the early marker of myocardial stress brain natriuretic peptide (BNP) was quantified. The primers used are listed in Table 2.

**Table 2:** Specific human primers used for RT-PCR.

**Target gene**	**Primer sequence 5^**′**^-3^**′**^**
18S_for	aaa cgg cta cca cat cca ag
18S_back	cct cca atg gat cct cgt ta
ERα_for	tcc agc acc ctg aag tct ct
ERα _back	gat gtg gga gag gat gag ga
ERβ_for	aga aga ttc ccg gct ttg tg
ERβ_back	gcc agg agc atg tca aag at
BNP_for	gct cct gct ctt ctt gca tc
BNP_back	gga ctt cca gac acc tgt gg
IL-1β_for	ctg tcc tgc gtg ttg aaa ga
IL-1β_back	ttc tgc ttg aga ggt gct ga
IL-6_for	aaa gag gca ctg gca gaa aa
IL-6_back	agc tct ggc ttg ttc ctc ac
IL-8_for	cag gaa ttg aat ggg ttt gc
IL-8_back	aaa cca agg cac agt gga ac
IL-10_for	gtg gag cag gtg aag aat gc
IL-10_back	cag atc cga ttt tgg aga cc
TNF-α_for	tgt gag gag gac gaa cat cc
TNF-α_back	cac att cct gaa tcc cag gt
TGF-β_for	cca gat cct gtc caa gct g
TGF-β_back	cct cct tgg cgt agt agt cg

*BNP, brain natriuretic peptide; ER, Estrogen receptor; IL, interleukin; TGF, tissue growth factor; TNF, tumor necrosis factor*.

### Statistical Analysis

Results are given as median and interquartile range (median, IQR). Data analysis was done using the Statistical Product and Service Solutions program, version 25 (SPSS®, IBM, USA). The non-normal distribution of data was verified, and non-parametric tests were applied. Correlations between two independent parameters were analyzed by using Spearman's rank correlation test, and the results were reported by Spearman's rank coefficient (*r*_s_). The Mann–Whitney *U*-test was used to compare the clinical and biological parameters in two different groups. *P* ≤ 0.05 were considered significant, while *p* < 0.1 indicated a tendency toward significance.

## Results

The female patients were younger than the male patients [4 years (12) vs. 9.9 years (10), *p* = 0.047].

### mRNA Expression of ERs and Genes Implicated in Inflammation, Growth, and Stress Response

The myocardial expression of mRNA coding for ERα and ERβ was detected in all patients, independently of gender, age, and achieved puberty, respectively. In the whole cohort, the expression of ERβ-mRNA was significantly higher than that of ERα-mRNA [4.89 (0.49) vs. 4.22 (0.40), *p* < 0.0001]. The myocardial expression of mRNA coding for BNP and for all tested cytokines was also detected in all patients and was not influenced by gender or age except that of TGF-β that was significantly higher in males than in females (*p* = 0.024) and correlated with age (*r*_s_: 0.490, *p* = 0.028). TGF-β-mRNA was also higher in patients who had achieved puberty than in the others (*p* = 0.047).

**Figure 1:**
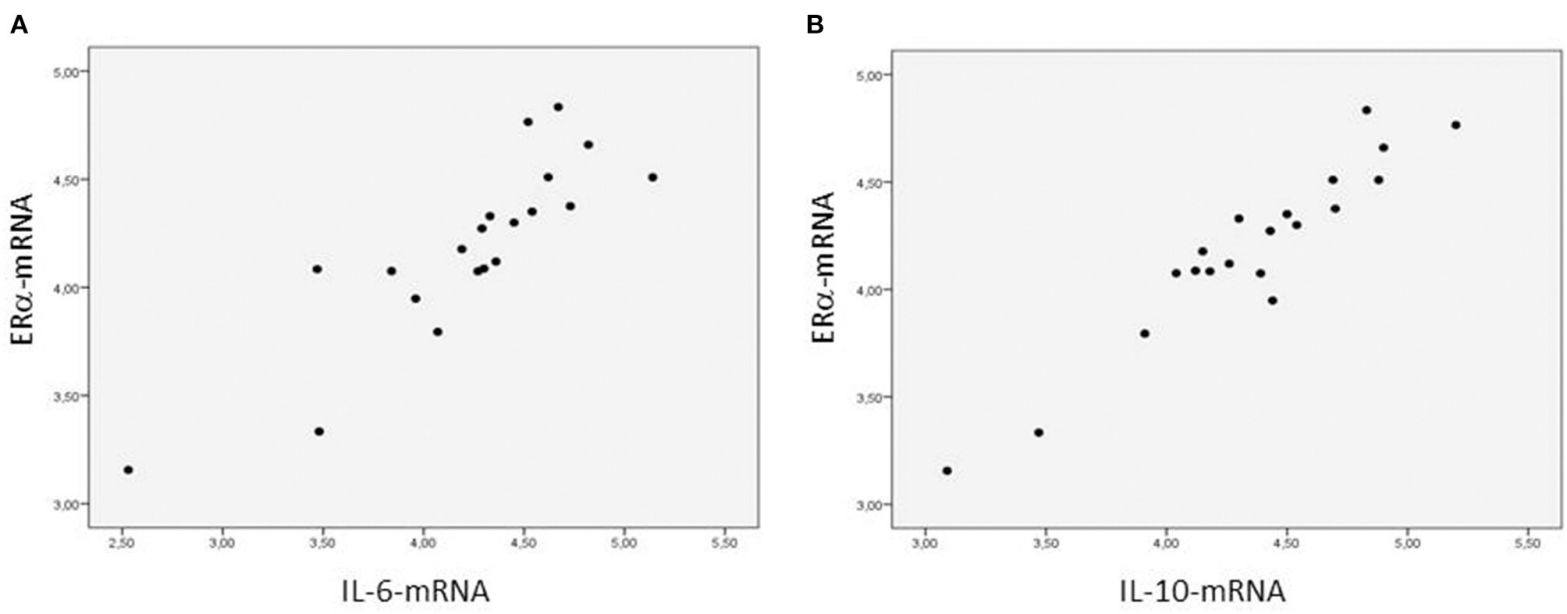
Relationship between the myocardial expression of ERα- and IL6-mRNA **(A)** and between the myocardial expression of ERα- and IL10-mRNA **(B)**. *N* = 20. Spearman correlation coefficient = 0.893 **(A)** and = 0.880 **(B)**, *p* < 0.0001, respectively. The mRNA expression of the target gene is corrected for that of 18S.

**Figure 2:**
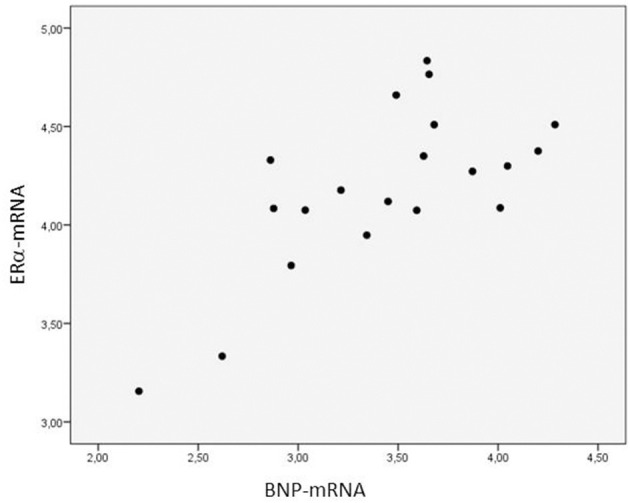
Relationship between the myocardial expression of ERα- and BNP-mRNA. *N* = 20. Spearman correlation coefficient: 0.930, *p* = 0.021. The expression of mRNA of the target gene is corrected for that of 18S.

The ERα-mRNA and ERβ-mRNA levels correlated with each other (*r*_s_: 0.922, *p* < 0.0001) and with the levels of mRNA coding for inflammatory cytokines (TNFα, IL-1β, IL-6, IL-8, and IL-10), growth factors (CT-1 and TGF-β), and the marker of myocardial stress (BNP), respectively (Table 3). Figure 1 shows the correlations between the myocardial levels of ERα-mRNA and IL-6-mRNA and between ERα-mRNA and IL-10-mRNA, respectively. Figure 2 shows the correlation between the myocardial levels of ERα-mRNA and BNP-mRNA.

**Table 3:** Correlation between myocardial expression of mRNA coding for ERα and for inflammatory cytokines, growth factors and early stress response genes.

	**ERα**	**ERβ**
IL-1 (*n* = 20)	*r_*s*_*: 0.892 *p:* 0.000	*r_*s*_*: 0.919*p:* 0.000
IL-6 (*n* = 20)	*r_*s*_*: 0.893 *p:* 0.000	*r_*s*_*: 0.803*p:* 0.000
CT-1 (*n* = 20)	*r_*s*_*: 0.926 *p:* 0.000	*r_*s*_*: 0.881*p:* 0.000
IL-10 (*n* = 20)	*r_*s*_*: 0.880 *p:* 0.000	*r_*s*_*: 0.850*p:* 0.000
TNF-α (*n* = 19)	*r_*s*_*: 0.832 *p:* 0.000	*r_*s*_*: 0.846*p:* 0.000
BNP (*n* = 20)	*r_*s*_:* 0.636 *p:* 0.003	*r_*s*_:* 0.525*p:* 0.018
TGF-β (*n* = 20)	*r_*s*_*: 0.890 *p:* 0.000	*r_*s*_*: 0.842*p:* 0.000

*r_s_, Spearman rank correlation coefficient. BNP, brain natriuretic peptide; CT, cardiotrophin; ER, estrogen receptor; IL, interleukin; TNF, tumor necrosis factor; TGF, tissue growth factor*.

### Correlation Between ER-mRNA Expression and Outcome Variables

The outcome variables were not different in males than in females except the creatinine concentration at 4 and 24 h post-operatively that was lower in females (*p* = 0.007 and *p* = 0.01, respectively). The outcome variables were not correlated with age except the creatinine concentration at 4 and 24 h post-operatively (*r*_s_: 0.812 and 0.804, respectively; *p* < 0. 0001, respectively).

**Figure 3:**
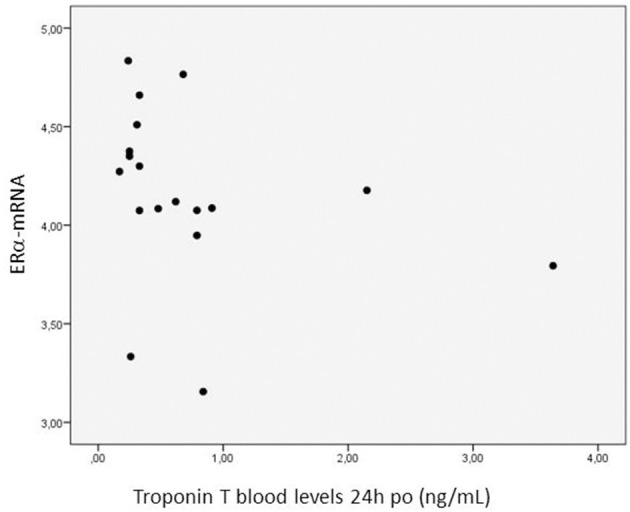
Relationship between the myocardial expression of ERα-mRNA and troponin T blood levels 24 h post-operatively. *N* = 18. Spearman correlation coefficient: −0.505, *p* = 0.032. The expression of mRNA of the target gene is corrected for that of 18S.

The expression of ERα-mRNA correlated negatively with the troponin T concentration at 24 h after the operation (*r*_s_:−0.505, *p* = 0.032) (Figure 3), positively with the ratio PaO_2_/FiO_2_ calculated at 4 h post-operatively (*r*_s_: 0.453, *p* = 0.059), negatively with the AST levels measured 24 h post-operatively (r_s_: −0.489, *p* = 0.047), and negatively with the water balance at 4 h post-operatively (r_s_: −0.487, *p* = 0.048).

**Figure 4:**
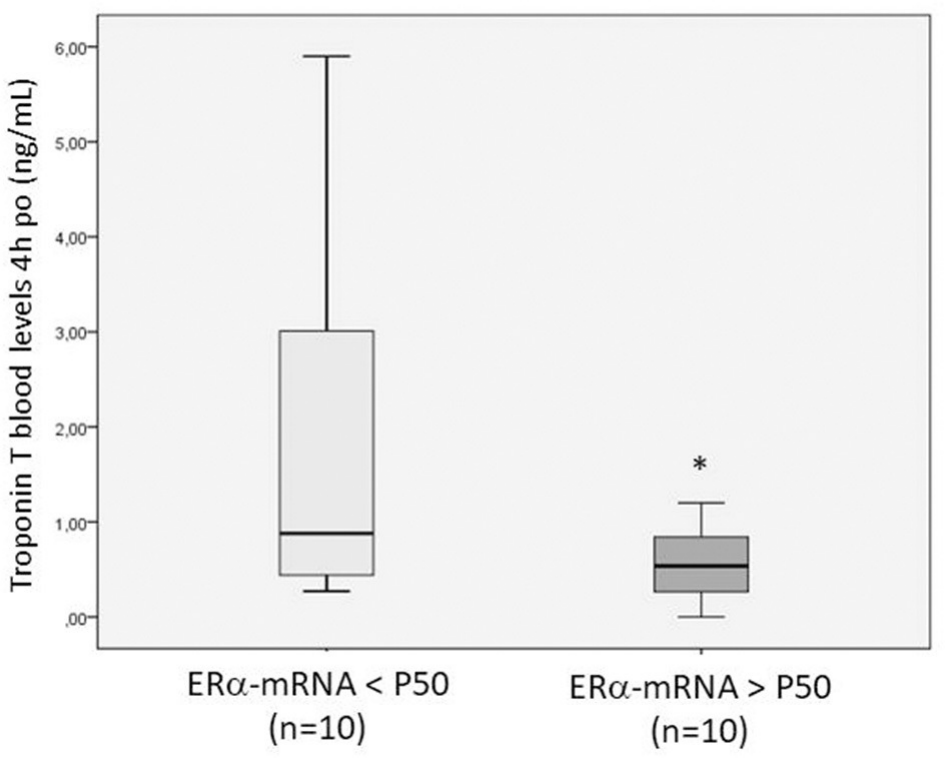
Box plot showing the troponin T blood levels measured 4 h post-operatively in patients with ERα-mRNA expression <P50 (light gray) or >P50 (dark gray). The box plot summarizes the minimal, maximal, and median (P50) values and interquartile range. *p = 0.043 between both groups.

The patients were divided in two groups (*n* = 10 each) depending on whether their levels of ERα- and ERβ-mRNA expression was greater or less than the median value (percentile, P, 50) and were compared to each other with respect to the post-operative outcome variables. The patients with ERα- and ERβ-mRNA expression >P50 showed lower troponin T levels at 4 and 24 h po than the others [0.53 ng/ml (0.69) and 0.28 ng/ml (0.09) vs. 0.88 ng/ml (2.69) and 0.79 ng/mL (0.78); *p* = 0.003, respectively] and lower AST concentrations at 4 and 24 h po [51.5 IU/L (19.7) and 58 IU/L (14); *p* = 0.043, respectively]. Figure 4 shows the troponin T levels in patients with ERα-mRNA expression less than or greater than P50.

## Discussion

### Myocardial Expression of mRNA Coding for ERs, BNP, and Cytokines

We show for the first time that mRNA coding for ERα- and ERβ is expressed in the right atrium of children and young adults with congenital heart defects independently of age, gender, and achieved puberty, respectively.

We also confirm our previous results demonstrating the myocardial expression of mRNA coding for pro- and anti- inflammatory cytokines, factors regulating cell growth and fibrosis, and proteins involved in the early cellular stress responses as a consequence of the activation of inflammatory pathways by hemodynamic overload (14). In this series, the expression of TGF-β increased with age, pointing to the importance of the duration of hemodynamic load on maladaptive myocardial remodeling and myocardial fibrosis (15).

Furthermore, the concentrations of mRNA coding for the different proteins tested correlated well with each other, suggesting an interplay between ERs and the mediators of inflammation, growth, fibrosis, and stress response of myocardial cells and therefore the participation of ERs in the complex mechanisms of myocardial remodeling in patients with congenital cardiac disease (13). In particular, the relationship between the expression of ER-mRNA and BNP-mRNA might indicate a role of hemodynamic overload in the upregulation of ERs as it has been shown in adult patients with aortic stenosis (12). Moreover, the fact that healthy women have higher BNP plasma levels than healthy men of the same age group suggests that estrogens may induce BNP expression throughout ER signaling (16). This is supported by the observation of increasing BNP blood levels after estrogen replacement therapy (17).

Our results show further that the mRNA expression of ERs correlated positively with the mRNA expression of pro- and also of anti-inflammatory cytokines. This apparent contradictory result might be explained by the complexity of the induction of inflammatory cytokines by the mechanical stimulation of cardiomyocytes. Indeed the early stress response leads to a sustained induction of pro-inflammatory cytokines that, in turn, initiates the gene expression of anti-inflammatory cytokines (14). Besides this, ERs are involved in the modulation of the inflammatory response by estrogens (18) and either activate or repress gene expression depending on local estrogen concentrations (19). Thus, higher estradiol (E2) levels downregulate pro-inflammatory cytokines such as TNF-α and upregulate anti-inflammatory cytokines such as IL-10 in different cell types, whereas low E2 levels stimulate TNF-α and IL-1β expression (20). The anti-inflammatory effect of E2 might be related to ERα that blocks TNF-α-induced IL-6 synthesis by interfering with nuclear factor kappa B (NFκB) (21). ERs inhibit multiple NFκB pathways and are therefore considered anti-inflammatory proteins (22–26).

The mechanisms by which low and high physiological concentrations of estrogens differentially affect ER activity to influence the expression of inflammatory genes are unclear. One possibility is that the low and high levels of E2 induce distinct transcriptional complexes and that this activates different pathways to promote or dampen inflammation (27). Our observation that the expression of ER-mRNA was not related to gender, age, or achieved puberty and that it was present in very young infants suggests an E2-independent mechanism for the activation of ERs in the myocardium of children. Indeed a large number of substances are likely to bind ERs such as xenoestrogens, in particular, phytoestrogens present in a wide spectrum of food constituents (28).

In our series, the myocardial concentrations of ERβ-mRNA were higher than those of ERα-mRNA. While a differential expression of both ERα and ERβ in the human right atrial myocardium has not been described that far, animal studies performed in neonatal and adult female rats have shown higher ERα-mRNA concentrations in the oldest animals (29). This suggests an influence of age on ERα expression in animal cardiomyocytes. In our patients, however, ERα expression was not age dependent. The role of inflammatory cytokines induced in the myocardium by hemodynamic overload on the upregulation of ERβ and down-regulation of ERα as has been documented previously (30, 31) remains speculative in our patient cohort who was heterogeneous in terms of quality and severity of cardiac defects and hemodynamic overload (14).

Besides this, hypoxemia could also have impacted ER expression as demonstrated in human breast cancer cell lines where hypoxia represses ERα (32).

Both the repression of ERα and the upregulation of ERβ involve the activation of hypoxia-inducible factor (HIF)-1α (33, 34) that is increased in the myocardium of children with cyanotic congenital cardiac disease, as we have shown previously (35). The putative influence of pre-operative hypoxemia on ER expression in the myocardium of patients with congenital cardiac disease was not investigated in our study, owing to the fact that only three patients of this series were cyanotic.

### Impact of Myocardial ER-mRNA Expression on Post-operative Outcome

The secondary objective of this study was to address the question of whether myocardial ER expression may provide cardio-protection to patients undergoing cardiac surgery for congenital cardiac disease and be related to better post-operative outcome.

Our results showing that a higher expression of ERα-mRNA was associated to lower myocardial damage, improved lung function, lower water retention, and lower cytolysis in the early post-operative period might support the assumed protective role of ERs in this particular patient population (22–26). The reason why the expression of ERβ-mRNA did not correlate significantly with the outcome variables but with reduced fluid retention might be related to the statistical rank correlation analysis performed on the small patient group.

Open cardiac surgery in adults and children is associated with a systemic inflammatory reaction that relates to post-operative myocardial cell damage and multiple organ dysfunction syndrome being a severe issue (36). In this context, troponin release correlates with the importance of systemic inflammation, in particular, with the levels of circulating IL-6 (37). Inflammatory proteins such as complement proteins are present in the circulation immediately after connection to the extracorporeal circuit and initiate the synthesis of pro- and anti-inflammatory cytokines by circulating and tissue cells (38). An adequate anti-inflammatory balance is thought to be necessary to limit and/or terminate inflammation and protect from organ injury (39).

The anti-inflammatory potential of myocardial ERs may provide peri-operative organ protection against operative and inflammatory stress. In an experimental sepsis model classically associated with a systemic inflammatory reaction, ERβ agonists provide increased survival and reduced tissue damage and modify the genomic sepsis signature with a decreased expression of pro-inflammatory genes (40). Besides these nuclear-mediated effects of ERβ, ERα initiates the activation of acute protective pathways *via* non-nuclear mechanisms involving the activation of kinases that enhance the phosphorylation of eNOS, PI3K/Akt, and ERK1/2 (41, 42).

While our results, taken together, might indicate that gender- and age-independent expression of ERα and ERβ in the myocardium of young patients undergoing cardiac surgery works as protective, more experimental data are needed to answer the question of whether the modulation of ER expression in the myocardium would participate to improve peri-operative organ protection in this patient group. For this purpose, an animal model of cardiac surgery for congenital cardiac disease with hemodynamic overload (15) involving pre-operative induction of ERs by pharmacological or genetic engineering procedures should be established.

### Limitations

Our study has several limitations. The small number of patients investigated and their heterogeneity in terms of cardiac diagnosis did not allow us to analyze the role of the quality of hemodynamic overload and of the degree of hypoxemia on ER-mRNA expression.

Furthermore, the limited size of the myocardial samples was insufficient to quantify protein synthesis of our target genes and prejudge the biological activity of ER induction.

Finally, we describe an association between higher myocardial ER-mRNA expression and lower clinical and biological markers of post-operative organ damage but are not able to give evidence of the organ-protective role of ERs during cardiac surgery for congenital cardiac defect at this stage. This would require experimental studies involving the modulation of ER expression in a model of cardiac surgery for congenital cardiac disease.

## Conclusion

Our study shows, for the first time, that ERα and ERβ are expressed at the mRNA level in the myocardium of young patients with congenital cardiac defect independently of gender, age, or puberty. The correlation between ER-mRNA expression and that of pro- and inflammatory cytokines, growth factors, and early stress response genes suggests an interplay between inflammatory and ER-activating pathways. The association between a higher ER-mRNA expression and lower clinical and biological markers of post-operative organ damage might indicate a protective role of ER pathways in the setting of cardiac surgery for congenital cardiac disease.

## Data Availability Statement

The raw data supporting the conclusions of this article will be made available by the authors, without undue reservation.

## Ethics Statement

The studies involving human participants were reviewed and approved by Ethics Committee of the Aachen University of Technology. Written informed consent to participate in this study was provided by the participants' legal guardian/next of kin.

## Author Contributions

HR: data analysis and manuscript redaction. NF: data collection and data analysis. RH: data collection and study design. JV-J: data collection and manuscript revision. A-SP: study design and manuscript revision. M-CS: study design, manuscript redaction and revision. All authors contributed to the article and approved the submitted version.

## Funding

This work was supported by a grant of the University Hospital Liège (FIRS) to M-CS.

## Conflict of Interest

The authors declare that the research was conducted in the absence of any commercial or financial relationships that could be construed as a potential conflict of interest.

## Publisher's Note

All claims expressed in this article are solely those of the authors and do not necessarily represent those of their affiliated organizations, or those of the publisher, the editors and the reviewers. Any product that may be evaluated in this article, or claim that may be made by its manufacturer, is not guaranteed or endorsed by the publisher.

## Abbreviations

AST: aspartate aminotransferase
BNP: brain natriuretic peptide
CPB: cardio-pulmonary bypass
CT-1: cardiotrophin-1
DNA: desoxyribonucleic acid
E2: estradiol
eNOS: endothelial nitric oxide synthase
ER: estrogen receptor
ERK1/2: extracellular signal-regulated kinases
FiO_2_: inspired oxygen fraction
HIF: hypoxia-inducible factor
IL: interleukin
IQR: interquartile range
mRNA: messenger ribonucleic acid
P50: percentile 50
PaO_2_: partial arterial oxygen pressure
PCR: polymerase chain reaction
PI3K/Akt: phosphatidylinositol 3-kinase/protein kinase B
TGFβ: tissue growth factor-β
TNFα: tumor necrosis factor-α.
